# Differences in Consumption Behaviour of Dietary Supplements in Competitive Athletes Depends on Sports Discipline

**DOI:** 10.3390/nu16030374

**Published:** 2024-01-27

**Authors:** Eduard Isenmann, Pia Tolle, Stephan Geisler, Ulrich Flenker, Patrick Diel

**Affiliations:** 1 Department of Molecular and Cellular Sports Medicine, Institute for Cardiovascular Research and Sports Medicine, German Sports University Cologne, 50933 Cologne, Germany; piatolle94@googlemail.com (P.T.); u.flenker@dshs-koeln.de (U.F.); diel@dshs-koeln.de (P.D.); 2 Department of Fitness and Health, IST-University of Applied Sciences, 40233 Dusseldorf, Germany; sgeisler@ist-hochschule.de

**Keywords:** dietary supplements, consumption behaviour, sports categories, dietary categories

## Abstract

Background: The consumption of dietary supplements (DS) is widespread among the general population and competitive athletes. However, only a few competitive athletes seek information from experts about the effects and use of DS. Furthermore, it is currently unknown whether certain sports have a higher affinity for DS than others. Methods: This study aimed to identify differences between different sports categories and subgroups that may have a very high affinity for DS. For this purpose, competitive athletes were surveyed. The survey included the type of sport, the training frequency, the number of competitions, the consumption behaviour of five DS categories (general health, regeneration promotion, performance enhancement, booster, and weight loss) as well as personal data such as biological sex and age. Subsequently, correlations, configural frequencies (CFA), and multiple correspondence analyses (MCA) were used to identify subgroups with a high affinity of consumption behaviour. Results: A total of 409 questionnaires could be evaluated. It was found that all DS categories except weight loss were related. In addition, it was observed that in sports from the power category and from the endurance category, there was even higher consumption behaviour than in other sports categories. Male power athletes in particular have a higher affinity for consuming DS than other subgroups. Conclusions: This study shows that there is a clear different consumption behaviour depending on the type of sport. Male power athletes in particular are the subgroup with the greatest consumption behaviour and therefore require special education on the effects and use of DS. This subgroup in particular should receive increased attention in counselling on DS to minimise the possible risks of DS use.

## 1. Introduction

Dietary supplements (DS) are often associated with a healthy lifestyle and are therefore increasingly consumed [1]. The popularity of DS has been steadily increasing for more than a decade. While 64% of the population in the USA consumed DS in 2008, this figure had already risen to 80% in 2021 [1]. In this context, 98% of those surveyed reported taking vitamins and minerals as well as specific DS such as omega-3 fatty acids or probiotics (49%) and herbal botanicals (39%) [1]. DS with a specific sport context, such as protein shakes, amino acids, or creatine, were consumed by 29% of the respondents, according to their statements. In European countries such as Denmark, the Netherlands, Sweden, Germany, and the United Kingdom (UK), an increasing consumption of DS can also be observed [2,3,4,5,6,7]. The latest consumer surveys in the UK show that the number of consumers has risen by 19% to 20 million since 2019 (2019: 16.5 million) due to the COVID pandemic (2020). Currently, 71.2% of respondents regularly consume food supplements, and 47.6% of these even consume at least one food supplement every day. The consumption of vitamin D (63%) and vitamin C (39%) in particular are the most frequently used food supplements. According to the latest surveys, more than three quarters (77%) of respondents in the 18–24 age group consume at least one food supplement [7]. A similar trend has been observed in Germany. Regular consumption behaviour increased from around 50% to more than 70% of those surveyed [5,8]. Motives for DS use are diverse, but ‘overall health/wellness’, ‘fill nutrient gaps in diet’, ‘energy provision’, and ‘immune health’ have most commonly been reported [1,9,10,11].

In competitive and performance sports, DS are also playing an increasingly important role in the daily training routine of athletes. A large number of studies have shown that despite the risk of contaminated supplements, which can lead to a positive doping test [12,13], athletes are not averse to consuming supplements regularly [14,15,16,17,18,19,20,21]. Research showed that 182 of 203 Division 1 athletes surveyed in the USA regularly consumed DS [22]. Similar consumption patterns can also be observed globally among competitive and elite athletes. In numerous studies, more than three-quarters of the athletes in a survey stated that they had consumed DS in the previous weeks and months [6,14,15,16,17,18,19,20,21,23]. In addition to competitive sports, an increased consumption of DS can also be observed among gym members. Studies in the USA show that almost 85% of fitness studio visitors consume food supplements [9]. A large amount took, at least five times a week, multivitamin/mineral supplements (MVM; 45%), protein shakes/bars (PRO; 42.3%), vitamin C (34.7%), and vitamin E (VE; 23.4%). A similar consumption behaviour was also observed in Italy in a survey of 316 gym owners (*n* = 89) and members (*n* = 227). The results showed a high prevalence of DS use in the population, with 85.4% of participants stating that they use DS, with great heterogeneity in the number and combinations used [24]. A study from Switzerland came to similar conclusions. The authors found that 82% of the 417 participants surveyed used at least one DS per week [25].

Concerning sexes, no difference was observed in the frequency of use [12,26]. However, individual supplements, such as creatine or protein supplements, which serve to enhance performance and increase muscle mass are primarily consumed by male athletes. In contrast, increased iron consumption is reported in female athletes [12,26]. However, it appears that performance level and training frequency have a greater influence on consumption patterns than gender. The higher the performance level and the number of training sessions per week, the more frequently DS are consumed [12,26]. The same relationship could also be found for age and supplementation of DS [12,26]. Studies investigating specific patterns of DS consumption concerning the DS category, such as whether certain DS are frequently consumed together or even only by certain athletes from specific sports, have not yet been investigated in detail.

Independent of this analysis, a statement by the International Olympic Committee (IOC) and the International Society of Sports Nutrition (ISSN) reported that only a few DS have a scientifically substantiated effect on the ability to perform and regenerate in sports [27]. DS which can enhance physical performance are primarily caffeine, creatine, nitrates, beta-alanine, and sodium bicarbonate [27,28,29,30,31]. Complex proteins and individual amino acids such as L-leucine, however, have only a trivial-to-small effect on increasing performance and muscle mass [27]. Regardless of the evidence on efficacy, supplements are often consumed without adequate education and advice from physicians, sports nutrition coaches, or scientists. Studies have shown that only one-sixth to one-fourth of athletes took advantage of detailed counselling to assess possible effects and risks [25,32,33]. Instead of consulting experts or professionals in the field of sports nutrition, athletes prefer to rely on coaches, friends, teammates, or family for information on consuming supplements [15,20,21,34]. Investigations have shown that athletes and coaches have significant differences in their knowledge of the effects of DS [35]. However, in general, they have clear deficits in knowledge of the mechanisms of DS and its actual use [35].

Although the use of DS and its benefits and risks were extensively studied, consumption behaviour and motivation have not yet been investigated, depending on the sports discipline. Based on a large number of doping cases in very specific sports that are either very endurance-based, such as cycling or middle- and long-distance running, or strength-based, such as weightlifting, it can be assumed that the use of DS in certain sports disciplines will be significantly higher than in others.

Therefore, this study aims to determine the consumption and motivational behaviour of dietary supplements depending on the type of sport. Furthermore, we investigate whether there are correlations between different DS categories such as DS for general health, regeneration, performance enhancement, or weight loss. We will investigate whether exclusively athletes of certain disciplines consume specific DS. For this purpose, competitive and elite athletes from Germany were surveyed.

## 2. Materials and Methods

### 2.1. Data Collection and Exclusion Criteria

All participants were informed about the study design and the project before the survey started and agreed to participate in the survey. All data were anonymized and complied with the Declaration of Helsinki. The consumption of DS was determined using an online questionnaire. The questionnaire was accessible via the online platform SoSci Survey for 28 days. After the questionnaire was uploaded online, the link was sent to the performance bases in Heidelberg, Cologne, and Berlin after consultation with the responsible persons. At the same time, competition athletes and sports nutrition coaches in Cologne, Heidelberg, Berlin, and Hamburg were personally contacted via email, Facebook, and Instagram. In addition, the questionnaire was distributed to other athletes via the snowball principle. 

All voluntary participants who regularly take part in national and international competitions were included in the survey as competitive athletes. All information on performance level was verified. Athletes who do not regularly participate in national competitions were not considered in the evaluation. 

### 2.2. Questionnaire

The questionnaire was previously tested in a pilot study according to the guidelines of Bühner et al. [36] and orientated to previous surveys [20]. This served mainly to assess possible areas of consumption and thus derive a meaningful evaluation.

The data was acquired through a questionnaire which encompassed 142 items in total. The questionnaire comprised three sections:

1. General information (e.g., biological sex, age, weight, professional activity);
2. Information on the type of sport performed (e.g., type of sport, training frequency per week, training hours per week, number of competitions, etc.);
3. Information on DS consumption behaviour (e.g., time of intake, number of capsules, bars, or pills per day).

Training volume and sports category served as independent variables. Training volumes were estimated as a combination of self-reported training frequencies [1/week] and of self-reported training lengths [h/week]. Sports categories were defined arbitrarily as follows:

- Combat sports (Combat): e.g., boxing, judo, etc.
- Endurance sports (Endur): e.g., long-distance running, cycling, triathlon, etc.
- Power sports (Power): e.g., weightlifting, powerlifting, but also climbing/bouldering.
- Team and racket sports (Team/Rack): e.g., football, tennis, etc.
- Technical sports (Tech): e.g., acrobatics, gymnastics, competitive dancing, etc.
- Miscellaneous (Misc): sailing, equestrian, shooting, and other sports lacking sufficiently definable requirements.

Dietary supplements were predefined to fall into five categories which putatively serve the following purposes:

- General dietary support (“Diet”);
- Support of regeneration (“Reg”);
- Performance enhancement (“Perf”);
- “Booster” (“Boost”);
- Support of weight loss (“WtLoss”).

Furthermore, respondents could indicate several supplements for each category. Consequently, the questions on each supplement were answered separately. Each item was followed by several sub-items specifying consumption habits. However, the variables were employed on a binary scale, i.e., whether the subjects currently consume or did ever consume supplements, falling into the respective category (Yes|No).

If the question was answered with yes, the consumption behaviour was asked with a six-point ordinal scaling. One capsule/tablet/scoop per day to five capsules/tablets/scoops per day, or the no answer option applies. Subsequently, the continuity to supplementation was checked. The following questions were used in this regard:

- Do you currently consume the supplement (Yes|No)?
- How often per week do you consume this supplement?
- At what time do you consume this supplement (before|during|after training)?
- Do you consume the supplement continuously or only during certain phases (e.g., competition)?

### 2.3. Data Extraction and Analyses

All data collected were transferred from SoSci to the current version of Microsoft Excel(Version 2311). For the data analysis, the current libraries of R Statistics (see Supplementary Materials File S1) were used [37,38,39,40]. Consumption patterns were analysed using Configural Frequencies Analysis (CFA) and Multiple Correspondence Analysis (MCA) [38,41]. Possible associations of age, training volume, sex, and sports category with consumption patterns were investigated via the correlations of these variables with the MCA dimensions. The statistical methods generally follow approaches that fall into the scope of exploratory data analysis (EDA). MCA and CFA are explicitly categorised correspondingly. These techniques serve to discover and visualize patterns in the data. This approach is fundamentally entailed by the rationale of the study to detect consumption patterns of athletes. Significant differences were set at *p* < 0.05.

## 3. Results

A total of 623 people took part in the survey. The questionnaires of 409 people were included in the analysis. The remaining questionnaires were not answered completely or were abandoned early. In total, 215 (53%) persons stated that they belonged to the female biological sex and 194 (47%) to the male biological sex. No person was assigned to the affiliation divers or other. The participants were aged between 12 and 52. The distribution by age group is shown in Table 1.

The following sports were named and assigned to the primary sports category. The data of athletes who exclusively compete at national level (qualification for German championship) and international level (European championship, world championship, Olympic Games) were taken into account (Table 2).

**Figure 1:**
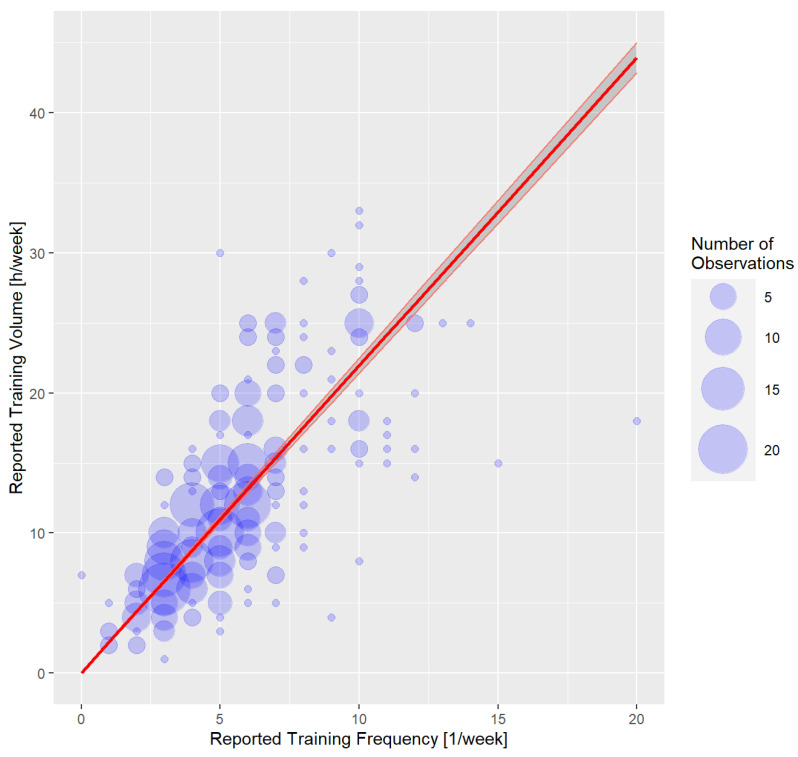
Bubble plot of reported training frequency and training volume.

Figure 1 shows the reported training frequencies and volumes of the respondents in a bubble plot. The training frequency ranged from 1 to 20 training sessions and up to 33 h per week.

Table 3 shows the absolute and percentage data on the general consumption of DS of the individual sports categories. It can be seen that consumption behaviour differs between the sports categories.

### 3.1. Sports Category and Dietary Consumption

**Figure 2:**
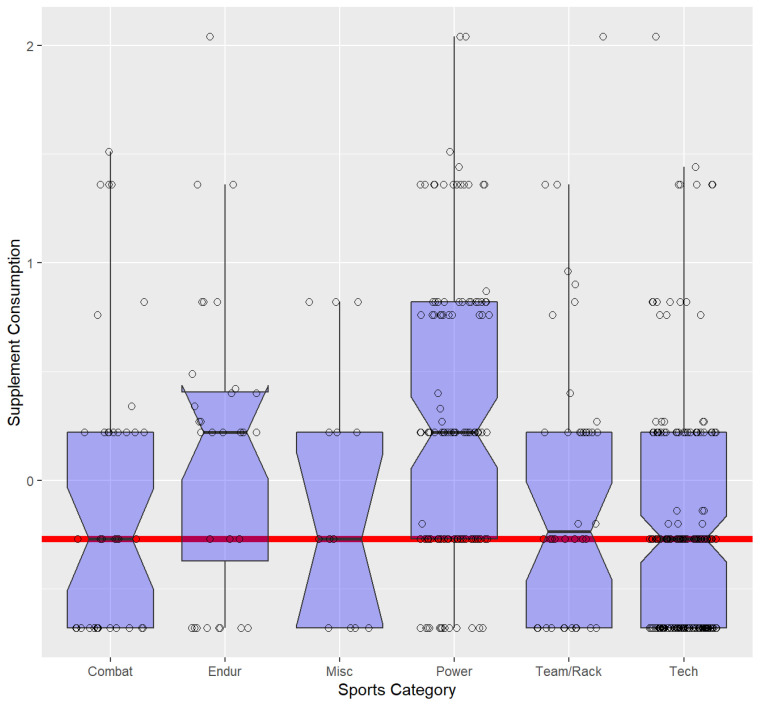
Boxplots of supplement consumption grouped by sports categories. The red line shows the median of all participants. Sports categories power and endurance have a higher median than the other categories.

Figure 2 shows a boxplot of supplement consumption grouped by sports categories. With the exceptions of endurance and power sports, the medians of supplement consumption categories are virtually identical to the grand median. Endurance and power sports exhibit medians above the grand median. In addition, both corresponding approximate 95% confidence intervals do not include the grand median. While this observation does not represent a strict testing procedure, it can be stated that endurance and power sports exhibit higher rates of supplement usage.

### 3.2. Correlation of Dietary Categories

Correlation analysis of the consumed supplement categories indicated that “WtLoss” represents a rather independent dimension. All other categories have a small to moderate correlation (Table 4).

### 3.3. Configural Frequencies Analysis of Dietary Category

**Figure 3:**
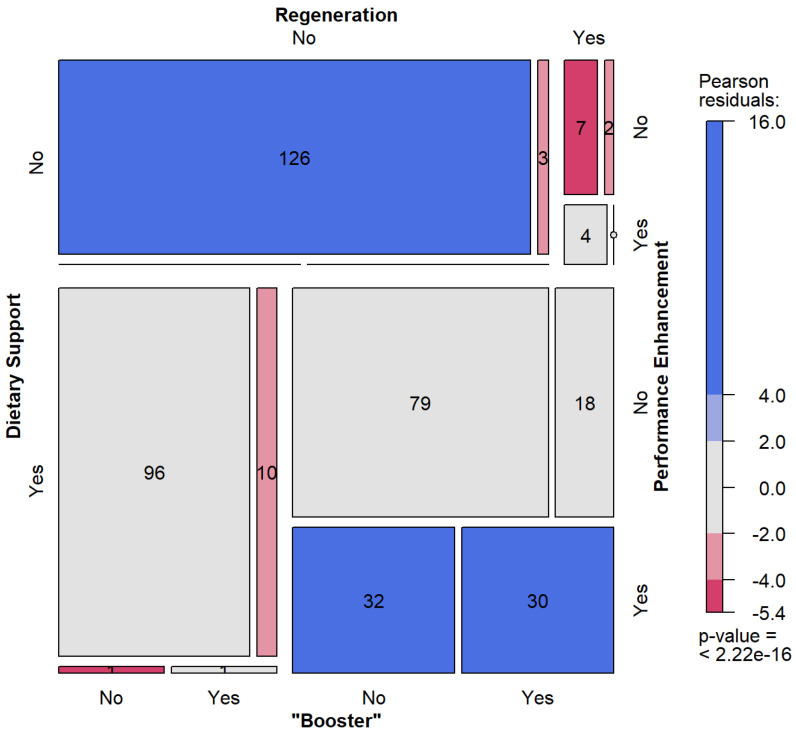
Mosaic plot for frequency analysis of the consumption of the supplement categories “Diet”, “Reg”, “Perf” and “Boost”. Each supplement category could be answered with yes or no. At the top left is the number of participants who answered no to all four categories. On the bottom right, all categories with yes. The grey colour means the actual number of responses is the same as the expected number of responses. Dark blue markers mean that significantly more responses were identified than expected. Dark red marks mean significantly fewer responses were identified than expected.

Configural frequency analyses showed that the consumption of “Diet” only (*p* < 0.01) and the combined consumption of “Reg” and “Boost” were significantly under-represented (*p* < 0.01) (red). The refusal of the consumption of any supplements was significantly over-represented (*p* < 0.001) as well as the combined consumption of “Diet”, “Reg”, and “Perf” (*p* < 0.05) (blue). The combined consumption of “Diet”, “Reg”, “Perf”, and “Boost” were likewise significantly over-represented (*p* < 0.001) (blue) (Figure 3).

### 3.4. General Association between Dietary Categories, Age, Sports Category, Sex and Training Frequency

**Figure 4:**
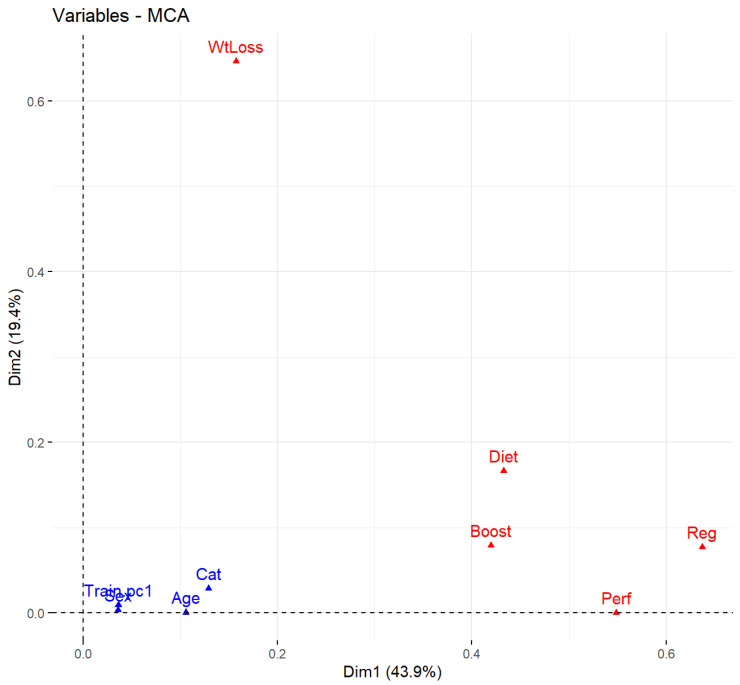
Multiple correspondence analyses (MCA) of supplements categories, training frequencies, sex, age, and sports category (cat). blue are general categories and red specific for supplement categories.

MCA confirmed that “Wtloss” consumption is mostly independent of that of the other categories. The remaining categories can be combined into a single dimension (Dim1) reflecting about 44% of the total variance. Dim1 most likely reflects general supplement consumption (GSC). GSC significantly depends on the sports category (cat) (*p* < 0.05). Higher age, higher training volumes, and sex are correlated with higher GSC values (Figure 4).

### 3.5. Interaction of Different Sports Categories, Sex, and Dietary Categories

**Figure 5:**
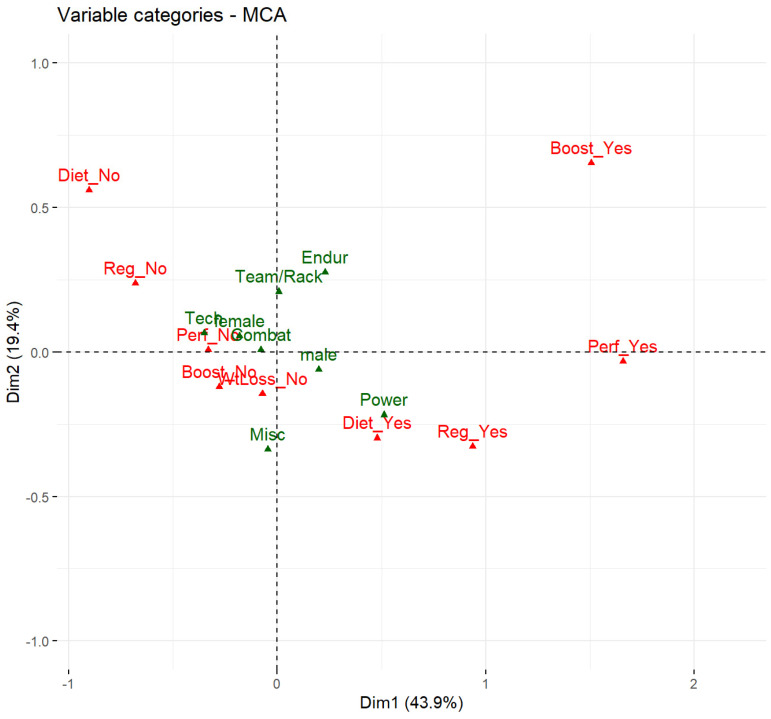
Interaction of different sports categories, sex, and dietary categories. Analysis shows that male strength athletes consume supplements from all categories (except WtLoss). Green are the categories of sex (male or female) and sport type (Power, Endur, Tech, Combat, Misc), red are dichotomous variable of the supplement categories (yes or no).

The GSC is strikingly high in power sports and very low in technical sports. The results clearly show that male strength athletes consume DS from all categories. It appears to be slightly elevated in endurance sports. Men tend to have significantly higher GSC values than women (*p* < 0.05) (Figure 5).

## 4. Discussion

This study aimed to identify possible subgroups that either have a very high affinity for DS or a very low affinity. There is hardly any data to identify subgroups with increased consumption behaviour in competitive DS sports. It was found that strength and endurance athletes tend to consume DS more than other athletes. In addition, two subgroups were identified that differed from the population. Male power athletes clearly tend to supplement DS more than other athletes from the categories “Diet”, “Reg”, “Boost” and “Perf”. Female athletes from technically compositional sports tend not to consume dietary supplements. Furthermore, the category “WtLoss” is independently compared to the other categories and has no association with the consumption behaviour of the other categories.

The results of the general consumption behaviour of competitive athletes are confirmed by observations of previous studies [14,15,16,17,18,19,20,21]. In general, competitive athletes have a high affinity for DS. On average, 75% of the athletes surveyed consume DS [12]. Similar to previous research from other countries, a correlation between consumer behaviour and age and training frequency was generally found [12,26]. It was found that the higher the training frequency and age, the more DS are consumed. It was also interesting to note that even the type of sport has a decisive influence on consumer behaviour. The results also indicate that in sports where only one or two conditional skills, such as strength and power (e.g., weightlifting, powerlifting, sprinting) or endurance (e.g., middle- and long-distance running or triathlon) dominate, there is a higher affinity for DS than in sports where technical and tactical components, such as tennis, soccer, gymnastics, or martial arts, has an important role. A similar consumption behaviour was also observed in some surveys of gym members [9,24,25]. Here, it was also found that consumption is even slightly higher than for sports in general. However, it cannot be assumed in this context that more DS are consumed in gyms in general. Some studies have not been able to determine this increased consumption [30,31]. In this context, it appears that the type of gym and the population exercising there have a decisive influence on the results. 

In addition to strength athletes, an increased consumption behaviour can also be observed among strictly endurance athletes. One possible reason could be that strength or endurance sports are primarily focused on the physiological dimension. Therefore, the consumption has to be put in the context of the performance level, the surveyed population, and their goal. The results suggest that in sports where the conditional skill of strength dominates, athletes tend to use supplements very heavily.

Another important finding is the correlation between the DS categories. All DS categories except WtLoss are interrelated. This has not been demonstrated in any previous study. It can be assumed that when DS for performance enhancement (such as creatine or phytosteroids) are used, DS from the “Reg” category (e.g., protein supplements) and the ”Diet” category (e.g., multivitamin supplements) are also used. This could even be confirmed with the CFA. Based on these, three subgroups were found to be overrepresented: one subgroup that does not consume DS and one that consumes from all categories (except WtLoss). The group that consumes from all categories must be differentiated again. DS from the “Boost” category is not used by all athletes. However, there is currently no comparable study that can explain or confirm this observation. Presumably, the motives for this category are different from those for the other three categories. However, these were not explicitly addressed in this study and should be examined again separately in the future.

However, the unsystematic consumption of many DS from multiple categories is not advisable for several reasons. As previous studies have shown, the use of DS can also lead to positive doping tests in elite athletes [13]. Consequently, the risk of a positive doping test may increase if a variety of DS is used. Furthermore, it is known that some dietary supplements can even be counterproductive for muscular adaptation when consumed in high doses. In particular, vitamin C and E, as well as alpha-lipoic acid, are currently being actively discussed regarding their potential to inhibit muscular adaptations [42,43]. Consequently, for competitive athletes and their coaches, it is crucially important to know in which phase of training they are currently and which dietary supplements they can use chronically in this phase without suppressing physiological and muscular adaptations. It is crucial whether competitive athletes are in the preparation, competition, or transition/regeneration phase [44]. As a third important aspect, the interrelationships between two or more dietary supplements have hardly been investigated. Consequently, it is currently not possible to estimate whether specific combinations scientifically exhibit an additive effect or which combinations may even inhibit each other. Additionally, the countless possibilities for combinations and the different dosages make it challenging to provide recommendations. Therefore, athletes are advised to minimize the number of different dietary supplements as much as possible [27].

Furthermore, this is the first study to use MCA to identify certain subgroups that mainly consume DS from all categories. In particular, male power athletes reported consuming supplements from all four categories at the same time. The number of DS in the individual categories was not explicitly evaluated, but only the general consumption behaviour. But it seems that special male power athletes, and not general male athletes from other sports categories, try in particular to optimise their performance with DS and maximise muscular adaptation processes. These findings are also consistent with previous studies showing that creatine or protein supplements tend to be consumed more by male athletes [12,26]. In contrast to previous studies, it was also found that other DS categories are increasingly consumed by male power athletes. It was found that dietary supplements (DS) from the “Diet” and “Boost” categories are also consumed more frequently by this subgroup. Because of this, these athletes need special education about DS. However, only a few athletes currently take advantage of expert advice and education on DS but rather rely on the experiences of their coaches, training colleagues, or self-researched information [25,34,35]. Furthermore, initial research also shows that females tend to use the services of experts more than males [25]. It is therefore crucial that, in general, athletes have more access to, and at the same time make use of, DS experts. Male power athletes in particular should be able to receive special care in terms of doping prevention. Current estimates suggest that about 6.4–8.8% of positive doping cases are due to contaminated DS [13]. Consequently, the subgroups that unknowingly consume a lot of DS are at increased risk. In addition, it is not possible to assess the interactions between the supplements and the possible side effects. Currently, there are hardly any studies that have investigated the interactions of several DS, so this cannot be estimated at present. Therefore, in addition to better educating athletes about dietary supplements, further systematic investigations of individual dietary supplements, as well as combinations of different supplements from the same category and different categories, should be conducted to identify potential additive or inhibitory effects, as well as potential side effects. Studies should primarily include athletes from competitive sports, taking into account the sport, training phase, and gender, to obtain detailed insights. Consequently, these findings can be used in advising athletes to enhance the quality of education.

## 5. Limitations

Besides the important new findings, this study also has some limitations. One aspect is that as in previous studies from different countries [5,14,15,16,17,18,19,20,21], only a small number of athletes could be reached. Consequently, the knowledge gained should only be considered in the overall context. Furthermore, no statement can be made regarding which exact supplements are used by which subgroups. Due to the large number of different DS in the individual categories and the large number of different providers for these, a subanalysis was not possible in this context. Another limitation of the study is that no exact dosage information was obtained for the individual DS so that the possible risks could be better assessed. However, it is generally difficult in quantitative surveys of athletes to obtain exact information on dosages. Moreover, the analysis did not additionally check the individual DS categories and how many DS were indicated at the same time, so no statement can be made in this context. Consequently, this should be taken into account in future surveys.

## 6. Conclusions

This survey examined potential differences in the consumption behaviour of dietary supplements (DS) based on the type of sport. It also investigated whether there is a correlation between the DS categories. This study aimed to identify potential subgroups with an increased affinity for DS and the specific types of DS. It is widely acknowledged that knowledge about DS is low among both athletes and coaches, and only a fraction of athletes collaborate with nutritionists in this field. It could demonstrate that male power athletes have a significantly higher affinity for DS than other athletes. Additionally, it was shown that the consumption of different DS categories such as “Diet,” “Perf,” “Reg,” and “Boost” is interrelated. This implies that DS consumption often follows the all-or-nothing principle. In contrast, female athletes in technically demanding sports consume significantly fewer DS than other athletes. Statements regarding potential combinations of DS preparations and their possible effects and side effects cannot be made. However, the results clearly indicate that male strength athletes especially should seek external guidance from nutrition experts to be informed about potential risks.

## Figures and Tables

**Table 1:** Distribution of participants according to age groups.

Age Group	Number
12–18 years	107 (26%)
19–25 years	174 (42%)
26–35 years	109 (27%)
>35 years	19 (5%)

**Table 2:** Distribution of participants according to sports categories.

Sports Category	Power	Endurance	Team and Racket	Combat	Technique Composition	Miscellaneous
Number (*n* = 409)	112	32	40	37	175	13
	Weightlifting Powerlifting Bodybuilding American Football Strongman Bouldering Climbing CrossFit High Jump Bar High Jump Long Jump Sprint	Triathlon Running (long- and middle-distance) Cross-country Skiing Mountain Biking Rowing Road Cycling Swimming Canoe Racing	Soccer Volleyball Basketball Handball Tennis	Boxing Karate Brazilian Jiu-Jitsu Judo Wrestling Thai Boxing Mixed Martial Arts (MMA)	Gymnastics Cheerleading Vaulting Acrobatic Trampoline Rhythmic Gymnastics Rhoenrad Gymnastics Dancing Water Diving	Sailing Sport shooting Horseriding
Training sessions/week	5.29 (1.62)	6.56 (3.3)	4.8 (2.15)	6.38 (3.78)	4.79 (2.25)	5.23 (2.77)
Hours of training/week	10.7 (4.08)	12.8 (5.96)	9.57 (5.08)	11.2 (5.67)	12.9 (7.16)	11.2 (5.87)
Numbers of Competitions	4.88 (5.49)	6.81 (6.12)	26.1 (21.5)	6.51 (6.34)	7.06 (4.25)	6.0 (6.1)

**Table 3:** Absolute and percentage data on the general consumption of DS depends on sports categories.

DS Consumption	Power	Endurance	Team and Racket	Combat	Technique Composition	Miscellaneous
Yes	99 (88.4)	24 (75.0)	28 (70.0)	23 (62.2)	104 (59.4)	9 (69.2)
No	13 (11.6)	8 (25.0)	12 (30.0)	14 (37.8)	71 (40.6)	4 (30.8)

**Table 4:** Multiple correlations of supplement categories.

	Diet	Reg	Perf	Boost	WtLoss
Diet	1.00	0.49	0.27	0.24	0.10
Reg	0.49	1.00	0.50	0.31	0.16
Perf	0.27	0.50	1.00	0.37	0.18
Boost	0.24	0.31	0.37	1.00	0.23
WtLoss	0.10	0.16	0.18	0.23	1.00

“Boost” = Booster; “Diet” = general dietary support; “Perf” = performance enhancement; “Reg” = support of regeneration; “WtLoss” = support of weight loss.

## Data Availability

The raw data can be viewed upon request.

## Supplementary Materials

The following are available online at https://www.mdpi.com/article/10.3390/nu16030374/s1, File S1: Current Versions of R library.

## Abbreviations

Boost	booster
Cat	category
CFA	configural frequency analyses
Combat	combat sports
Diet	dietary support
DS	dietary supplements
EDA	exploratory data analysis
Endur	endurance sport
GSC	general supplement consumption
MCA	multiple correspondence analyses
Misc	miscellaneous
Perf	performance enhancement
Power	power sports
Team/Rack	team and racket sports
Tech	technical sports
Reg	regeneration
WtLoss	weight loss
