# Supplementary material for: Differences in Consumption Behaviour of Dietary Supplements in Competitive Athletes Depends on Sports Discipline
Source: Nutrients. 2024 Jan 27;16(3):374. doi: 10.3390/nu16030374 (PMC10857381; doi:10.3390/nu16030374)
Supplement: Supplementary file 1 [file nutrients-16-00374-s001.zip › nutrients-2767227-supplementary.pdf]

Supplemental Materials

Current Versions of R libraries:

```
library("bookdown")
library("MASS")
library("cfa")       # "Configural Frequencies Analysis"
library("gplots")
library("lattice")
library("FactoMineR") # "Multivariate Exploratory Data Analysis
                # and Data Mining"
library("factoextra") # Extract and Visualize the Results of
                # Multivariate Data Analyses
library("Gifi")      # Multivariate Analysis with Optimal
                # Scaling
library("Factoshiny") # "Perform factorial analysis with a menu
                # and draw graphs interactively thanks to
                #'FactoMineR' and a Shiny application."
library("CAinterprTools") # Precisely does what the name
                   # suggests ;-)
library("vcd")       # "Visualisation of Categorical Data"
library("xtable")
```
